# Diet chemistry and cross-kingdom microbiota associate with black soldier fly larvae performance on regional side streams

**DOI:** 10.1186/s42523-025-00509-6

**Published:** 2026-01-08

**Authors:** Friscasari F. Gurusinga, Sabine Hurka, Daniel Kreft, Till Röthig, Andreas Vilcinskas, Dorothee Tegtmeier

**Affiliations:** 1 Branch for Bioresources, Fraunhofer Institute for Molecular Biology and Applied Ecology (IME), 35392 Giessen, Germany; 2 BMFTR Junior Research Group in Bioeconomy (BioKreativ) “SymBioÖkonomie”, 35392 Giessen, Germany; 3 LOEWE Centre for Translational Biodiversity Genomics (LOEWE-TBG), 60325 Frankfurt, Germany; 4 Institute for Insect Biotechnology, Justus Liebig University, 35392 Giessen, Germany

**Keywords:** Insect farming, Animal feed, Future protein, microorganisms, metaorganism, Microbial diversity, metabarcoding

## Abstract

**Background:**

Black soldier fly larvae (BSFL; *Hermetia illucens*) are increasingly farmed as a sustainable source of animal protein and are capable of converting diverse organic material into high-value biomass. Agricultural side streams represent an abundant and underutilized feed resource within the European Union (EU). However, their influence on BSFL development and gut microbial communities remains insufficiently characterized despite the central role of the microbiota in host nutrition, immunity, and health.

**Result:**

We evaluated five feed treatments, namely chicken feed (control), apple pomace, potato pulp (industrial-scale and lab-scale), and rapeseed cake, for their suitability in supporting BSFL growth from hatching to pupation. All feed treatments supported larval development, with time to pupation ranging from 25 days (rapeseed cake) to 87 days (potato pulp industrial-scale). Larvae reared on chicken feed exhibited significantly (7.6-fold) higher biomass compared to those fed with apple pomace, while the performance of BSFL reared on rapeseed cake was similar to that of the control larvae. Variations in development performance correlated with differences in feed nutrient profiles, particularly protein, fat, and fiber content. Furthermore, gut microbiota profiling via 16S rRNA and ITS2 amplicon sequencing revealed that gut microbial communities varied depending on diet chemistry. This was supported by functional predictions indicating an enrichment of fiber-degrading and nitrogen-fixing bacteria in larvae fed fiber-rich, nitrogen-poor diets. Notably, no methane production of L5 larvae was detected across treatments under tested laboratory conditions.

**Conclusion:**

This study demonstrates the potential of agricultural side streams to serve as viable feed substrates for BSFL production, with associated shifts in gut microbiota likely supporting host adjustment to less-optimal diets. These findings underline the importance of microbiota plasticity in BSFL digestion and highlight the relevance of feed composition in optimizing large-scale insect farming for sustainable protein production.

**Supplementary Information:**

The online version contains supplementary material available at 10.1186/s42523-025-00509-6.

## Introduction

The global population is estimated to reach 9.7 billion in 2050 [1]. According to recent data from the Food and Agriculture Organization (FAO), the global production of primary crops reached 9.9 billion tons in 2023. This represents a 3% increase since 2022 and a 27% increase since 2010, reflecting the rising global demand for food [2]. However, conventional animal farming already puts considerable pressure on natural resources, including land, water, and energy, while contributing to environmental degradation through greenhouse gas (GHG) emissions, deforestation, and biodiversity loss [3, 4]. In addition, both agriculture and aquaculture depend on high-protein feed sources, such as soybeans and fishmeal. The cultivation of soybeans is a leading driver of deforestation, particularly in South America, while fishmeal production contributes to the overexploitation of marine ecosystems [5–7]. This highlights the urgent need for alternative protein sources that are environmentally sustainable while supporting global food security.

Insect farming has emerged as a promising and sustainable alternative to protein production. It presents several environmental and practical advantages, such as short life cycles, minimal land use, reduced feed requirements, and reduced GHG emissions [8–11]. Several studies have demonstrated the use of insect protein as a viable alternative to fishmeal and soybean in poultry, pig, and aquaculture, which emphasizes the potential for the animal feed industry [12–18]. Despite these benefits, challenges such as consumer acceptance, regulatory frameworks, production scalability, and most importantly economic considerations remain critical barriers [19]. Currently, insect proteins are more expensive ($3,500–6000 per metric ton) than other traditional protein sources ($1,400–1800 for fishmeal and $400–500 for soybean) [20, 21]. Feed costs are a primary driver of insect farming expenses, presenting a promising option to improve economic viability [17, 22]. To reduce feed costs, insect farming may utilize industrial side streams, transforming low-value organic materials into high-quality protein. In addition to their economic advantages, these side streams offer the critical benefit of reducing competition between feed and food production and between different feed sources, compared to conventional livestock [23].

In the European Union alone, approximately 59 million tons of food side streams are produced annually, with a market value of €132 billion [24]. These side streams represent a considerable, often locally available feedstock for insect farming. Several side streams, including fruit pomace and vegetable pulp, often remain underutilized or discarded [25]. Oilseed press cakes, such as rapeseed and sunflower cakes, are produced in large quantities in regions with well-established oilseed processing industries. In fact, the total biomass of these by-products often exceeds that of the main product, accounting for roughly two-thirds of the output. This creates a challenge for oil mills as disposing of such large volumes would be costly thus making value-added uses particularly important. Given their high throughput at regional oil mills, stable composition, and co-location advantages (reduced transport, steady supply), oilseed press cakes represent a potential feed source for BSFL bioconversion, despite established livestock uses. Therefore, rapeseed cake should be considered because of its local abundance and process integration potential, rather than any claim of limited utilization [26–28].

The black soldier fly (BSF), *Hermetia illucens,* has gained attention as it can be used as a tool to valorize a wide range of organic waste into valuable protein and fat, thereby aiming to support a more circular and sustainable food system [29, 30]. The BSF larvae (BSFL) can be reared on a variety of low-value agri-food industry by-products and contain up to 50% protein and 35% fat [31–35]. In addition to their substrate flexibility, BSFL showed rapid growth, minimal water and land requirements, making them an economically interesting and environmentally sustainable option for protein production [10].

For an efficient digestion of organic materials, most insects rely on the presence of a symbiotic community of microorganisms within the gastrointestinal tract. These microbes play important roles in insect health and development, such as (i) support in digestion, nutrient absorption, and providing essential nutrients; (ii) detoxification of compounds; and (iii) regulation of the immune system [36–38]. For instance, the microbes produce enzymes to support the degradation of various organic materials, allowing efficient nutrient provision to the insect [36]. In addition, some insects like termites harbor symbiotic microbial communities that are specialized in the degradation of cellulose [39–41]. Cellulolytic microorganisms have also been isolated from BSFL and studied for their ability to break down fiber-rich compounds, suggesting a role in substrate digestion [42–44], and the addition of exogenous cellulolytic microbes can enhance substrate conversion by BSFL [45, 46]. Furthermore, microbial composition and diversity in the BSFL gut shifts dynamically depending on the feed type, likely supporting larval digestion [32, 38, 47, 48]. Therefore, the characterization of BSFL gut microbiota reared on different substrates can provide insights into host-microbe interactions.

In addition to facilitating the digestion and absorption of nutrients, the metabolic activity of insect’s gut-associated microbes may contribute to gaseous emissions. Wood- or detritus-feeding insects, such as termites, beetles, and cockroaches are mostly known to emit methane (CH_4_) [49–52]. Previous studies on methane emissions from BSFL have shown highly variable results, ranging from 0 to 10,006 mg/kg dry mass of larvae, depending on the composition and characteristics of the substrate [53–59]. Nevertheless, these emissions are substantially lower than those from conventional livestock systems, which contribute approximately 7.1 gigatons of CO₂-equivalent, accounting for around 14.5% of global anthropogenic GHG emissions. Cattle are largely responsible for a significant proportion of these emissions due to rumen fermentation [10, 60]. Thus, such comparison highlights the potential of BSFL farming as a climate-friendly, resource-efficient alternative for producing sustainable protein.

In this study, we use four different industrial side streams that are regionally available and low-cost in Germany, namely apple pomace, lab-produced potato pulp, industrial-scale potato pulp, and rapeseed cake to determine whether they support BSFL development and to characterize their impact on the gut-associated microbial communities. While previous BSFL microbiome studies have provided valuable insights into gut community composition, many have relied on a narrow set of substrates and lacked detailed nutrient profiling. In contrast, our study focuses on distinct, locally abundant agro-industrial side streams, integrating chemical characterization, larval performance, and cross-kingdom microbiota analyses. Before testing our hypothesis, we verified that these side streams show remarkable differences in their nutritional composition. We hypothesize that (1) the differences in the protein, fat, fiber and starch content of the side streams are reflected in the BSFL growth and composition, (2) cross-kingdom gut community composition varies between side streams but differ from the feed community due to host selection, and (3) no methane is detected in L5 BSFL under tested laboratory conditions, highlighting a potential sustainability benefit. Due to the ability of the gut microbiota to mediate various physiological processes, including digestion and nutrient absorption, dietary changes likely support specific microbial taxa that could be selected by the host or are better adjusted to the available substrates. By characterizing and comparing microbial communities across dietary treatments, we aim to identify diet-specific microbial trends and assess their potential functional contributions to host metabolic capacity.

## Materials and methods

### Experimental feeds

To assess the influence of industrial side streams on BSFL growth performance and gut microbial communities, five feed types were used as experimental treatments, namely chicken feed (CF), apple pomace (AP), rapeseed cake (RC), and two types of potato pulp: potato pulp lab-scale (PPL) and potato pulp industrial-scale (PPI). CF (GoldDott Eierglück, Raiffeisen, Muenster, Germany) was used as a control diet [33]. AP was sourced freshly from the apple wine industry (Kelterei Mueller, Butzbach, Germany). Prior to sourcing, Fraunhofer IVV (Freising, Germany) used the Kuras potato variety (Agrico, Emmeloord, Netherlands) to produce PPL by peeling (Flott 16K potato peeling machine, Flottwerk, Rotenburg a.d. Fulda, Germany), juicing (HR1921/20 centrifugal juicer, Philips, Amsterdam, Netherlands), and then extracting the protein and starch from the potato juice. The pulp was then stored in vacuum-sealed plastic bags at −20 °C until used as feed. PPI is a side stream from the industrial starch production (Südstärke GmbH, Schrobenhausen, Germany) and was delivered as a fresh product. PPI was produced from cleaned potato, were grated into shreds (Nivoba, Veendam, Netherland). Potato juice was separated from the shreds and starch grains in a decanter (Flottweg, Vilsbiburg, Germany). The solid phase from the decanter was collected and processed into pulp, resulting in the PPI feed. To reduce the moisture content for PPL and PPI, a portion of the pulp was dried at 50 °C (BDA-15 dehydrator, Beeketal Lebensmitteltechnik, Rastdorf, Germany) and ground (Spice and coffee EGK 200, Rommelsbacher, Dinkelsbühl, Germany). PPL and PPI were frozen and stored at −20 °C until experimental start. RC is a dry-pressed by-product from cold-pressed rapeseed oil production (Gültsteiner Mill, Herrenberg, Germany). The side streams were selected based on their abundant availability from German industries.

For experimental preparation, CF was milled (Mockmill 200 grain mill, Wolfgang Mock, Otzberg, Germany) as described in Tegtmeier et al. [33]. AP was dried at 40 °C (BDA-15 dehydrator, Beeketal Lebensmitteltechnik, Rastdorf, Germany) and ground (Thermomix TM6-1, Vorwerk Elektrowerke, Wuppertal, Germany). PPL and PPI were adjusted to a moisture content of 70–75% by mixing dried and wet pulp. Similar to CF, RC was also ground (Spice and coffee EGK 200, Rommelsbacher, Dinkelsbühl, Germany). Initial pH of each experimental feed was measured (pH-meter P21, OCS.tec, Neuching, Germany). Each feed’s moisture content was maintained at ~70% (TMT-MC-7828S soil moisture meter, Landtek Instruments Co. Ltd., Guangzhou, China) to ensure comparability until ≥50% of the larvae had reached the pre-pupae stage.

### BSFL stock colony

*Hermetia illucens* were provided by Bio.S Biogas (Grimma, Germany) and reared for over 35 generations continuously in our core-breeding at Fraunhofer Institute for Molecular Biology and Applied Ecology – Branch for Bioresources (Giessen, Germany). Adult *H. illucens* were kept in mesh cages (60 ×60 × 90 cm, Bioform, Nuremberg, Germany) in a greenhouse at 26 ± 1 °C, 60 ± 5% relative humidity, and a 12-hour light period was ensured using artificial LED light (3000 K L-PL-ECO623330, Lence Technology, Langen, Germany). Water was provided by water-soaked paper towels in a polypropylene box. Additionally, the cages were generously sprayed with water daily.

Eggs were harvested from the oviposition site (stacks of three wooded boards separated by washers) and placed in plastic boxes (19.5 ×16.5 ×9.5 cm) and sealed with lids. Each box contained 150 mg of eggs (~6,000 eggs) and was sprayed daily. The hatching rate varies from generation to generation ranging from 24 to 50%. Therefore, with 150 mg of eggs (~6,000 eggs per box), the initial number of hatched larvae per box was between 1440 and 3000. This resulted in a larval density between 4.47 and 9.3 larvae/cm^2^ in a plastic box measuring 19.5 × 16.5 ×9.5 cm. After ≥50% of eggs had hatched, the closed lids were replaced with fine mesh. At two-day interval, water and chicken feed were provided to the larvae *ad libitum* until they reached the prepupal stage. The larvae were kept in a climate chamber at 27 ± 1 °C and 65 ± 5% relative humidity in the dark [61].

### Experimental setup

In this study, three plastic boxes (19.5 ×16.5 ×9.5 cm) were prepared for each of the five feed treatments (total: 15 boxes). Egg clutches were collected from the core-breeding, placed in a plastic box (150 mg = ~6,000 eggs per box) and sealed with lids. The eggs used in this study were collected at the same time and derived from the same BSF generation. After ≥50% of the eggs in the same box hatched, the lid was replaced by fine mesh. The larvae were reared in a climate chamber at 27 ± 1 °C and 65 ± 5% relative humidity in the dark. The boxes were checked in two-day intervals and larvae were fed with experimental feeds *ad libitum* until ≥50% of the larvae reached prepupal stage. The larval growth was measured starting at a size of 3–4 mm (instar L3, 5–10 d), for which the age varied depending on the treatment. Based on BSFL availability, we assessed larval growth at two-day intervals by the averaging the weight of 25 randomly selected larvae for all treatments except for PPI and PPL, where 50 larvae from each of the three replicate plastic boxes were used (AT261 DeltaRange analytical balance, Mettler, Giessen, Germany). The pH of each of the five frass treatments was also measured (pH-meter P21, OCS.tec, Neuching, Germany).

### Feed and larval composition

To characterize the nutritional value of the insect feeds, we considered the content of dry matter, crude ash, crude fiber, crude fat, crude protein and starch. The composition of the BSFL was assessed based on their content of crude ash, crude fat, and crude protein. Approximately 20 g for each feed type and about 100 g L5 larvae for each treatment were separately ground with a mortar under liquid nitrogen and the moisture content was determined (M35 Moisture Analyzer, Sartorius, Göttingen, Germany). Samples were lyophilized for 72 h, then 1 g lyophilized feed and 0.5 g lyophilized larvae were analyzed for crude ash, protein, fat content, an additional 3 g lyophilized feed was used for crude fiber determination, and 0.1 g feed was used for starch content analysis. All feed and larval composition analysis were conducted in triplicates.

The crude ash content was determined by gravimetry. The samples were pre-incinerated in a quartz crucible over a Bunsen burner, incinerated twice for 6 h at 550 °C (L 9/11 muffle furnace, Nabertherm, Lilienthal, Germany), and the crude ash content was calculated using differential weighing. Crude fiber content was measured according to Scharrer-Kürschner [62]. The samples were homogenized, mixed with acetic acid mixture, and boiled for 30 min under the fume hood. The mixture was then filtered through ash-free filter paper and incinerated for an hour at 700 °C in an L 9/11 muffle furnace (Nabertherm, Lilienthal, Germany). The Weibull-Stoldt method was used to determine the crude fat content [62]. The samples were manually disintegrated for 30 min in 150 mL boiling 4 mol⋅L^−1^ HCl, then filtered, neutralized with hot water, and dried at 105 °C for 2 h. Fat was extracted automatically with *n*-hexane in a Soxtherm system (Gerhardt, Königswinter, Germany) and the content was determined gravimetrically. Crude protein was determined using the Kjeldahl method, which involves digesting the samples with sulfuric acid (Kjeldatherm, Gerhardt, Königswinter, Germany), automated steam distillation (Vapodest 500, Gerhardt, Königswinter, Germany) and titration (TitroLine 5000, SI Analytics, Mainz, Germany). The crude protein content was calculated based on a conversion factor of 6.25 [63]. Starch content was analyzed using Starch UV-method Kit (R-Biopharm AG, Darmstadt, Germany) according to the manufacturers protocol.

### DNA extraction

To assess gut microbial communities associated with BSFL from the different treatments (i.e., CF, AP, PPI, PPL, and RC), whole guts consisting of foregut, midgut, and hindgut were assessed. L5 larvae from generation 36 (CF, PPI, PPL, RC) with a weight of 100–150 mg were collected from each box and frozen at −20 °C. Due to the low weight of AP larvae, instar measurement is based on the diameter of the head capsule (0.58–1.04 mm) [64]. Next, the larvae were washed with autoclaved water and rinsed twice with 70% ethanol. Each larva’s gut was dissected under a stereomicroscope with freshly sterilized forceps. Next, DNA from individual dissected guts (nine replicates per diet) was extracted (NucleoSpin® Soil-Kit, Macherey-Nagel, Düren, Germany) according to the manufacturer’s protocol and including an additional initial bead beating step (FastPrep-24, MP Biomedicals, Solon, OH, United States) for homogenization and disruption of cells. Extracted DNA quality and quantity was verified (Take3 plate reader, BioTek Instruments, Winooski, VT, United States) as described in Tegtmeier et al. [33]. To ensure that the gut microbiota reflected the metabolically active stage of development rather than the transition to the prepupal stage, only actively feeding larvae were selected, based on criteria described in Tegtmeier et al. [33] and Barros et al. [64].

To compare microbial communities between sample groups (i.e., gut and feed), feed from all five types was considered at the initial feeding step, at L3-feeding, and at L5-feeding. Here, 100 mg of fresh feed samples at each time point was collected, and DNA was extracted according to the procedure described above (NucleoSpin® Soil-Kit). All extracted DNA was frozen and stored at −20 °C until further use.

### Microbial community analysis

Extracted DNA was sent to LGC Genomics (Berlin, Germany) for library preparation and sequencing. The primer pair U341F (5’-CCT AYG GGR BGC ASC AG-3’) and U806R (5’-GGA CTA CNN GGG TAT CTA AT-3’) [65] was used for the targeted amplification of the variable region V3–V4 of the 16S ribosomal RNA (rRNA) gene of bacteria and archaea. The fungal ITS2 region was targeted using the primers fITS7 (5’-GTG ART CAT CGA ATC TTT G-3’) [66] and ITS4 (5’-TCC TCC GCT TAT TGA TAT GC-3’) [67]. Sequencing was carried out on an Illumina MiSeq V3 platform (San Diego, CA, United States) generating paired-end reads with a read length of 300 base pairs. Samples were multiplexed and pooled for sequencing. After sequencing, demultiplexing, and removal of adapters and primers were performed by LGC Genomics using the bcl2fastq 2.20 software (Illumina, Inc., San Diego, CA, United States). Subsequent read analysis was conducted using QIIME2 2024.5 amplicon distribution [68]. ITS2 sequence reads were trimmed with the cutadapt plugin if the synthesized strand reached the second sequencing adapter [69]. The DADA2 plugin [70] was used for merging the 16S paired-end reads, while the ITS reads were not merged due to insufficient read pair overlap, where only the forward reads of the ITS2 target regions were used for further analysis. Subsequently, both datasets underwent filtering of chimeric sequences, and to infer amplicon sequence variants (ASV) using DADA2.

The RESCRIPt plugin with a patch corresponding to commit 839598b was used to construct a self-trained naïve Bayes classifier to apply for the taxonomic classification [71]. We used SILVA 138.2 [72] and UNITE 10 [73] to classify 16S rRNA and ITS2 data, respectively, both with a sequence identity threshold of 99%, respectively. Reference sequences from SILVA were trimmed to the target region [74]. For the ASV classification, a minimum confidence threshold of 0.7 was set for 16S and 0.94 for ITS2 [75]. To compile a stacked column chart, for each corresponding taxonomic hierarchy the following labels were set to N/A: NA, unidentified, uncultured, metagenom, Incertae_Sedis. The family assignments of 5 fungal genera (21 ASVs) have been manually renamed to their validly published families according to the NCBI taxonomy [76]. In addition and where necessary, higher taxonomic levels were updated (Table S3). A second classification step was performed on ASVs that were still unclassified. For this, a BLASTN 2.16.0 search (word_size 11, evalue 0.05, max_target_seqs 10) was performed against the preformatted nt BLAST database from NCBI (Aug 7, 2024 2:22 AM, downloaded August 13, 2024) [77, 78]. Results were selected manually based on the following two criteria: first, non-target ASVs (e.g., insects, plants, chloroplasts, and mitochondria) were discarded. Second, we updated the kingdom-level classification if the underlying BLAST hits had a sequence identity greater than 80% (alignment > 140 bp) and marked changes with a confidence of −1. All other ASVs were classified as unassigned and discarded. Unwanted ASVs (unclassified and non-target like mitochondria, chloroplasts) were removed from the datasets and, of note, no archaeal sequences were identified. Classification and calculation of relative abundances were based on unrarefied data. Alpha and beta diversity (species richness, Pielou’s evenness, Shannon index) and UniFrac distance matrix [79] were calculated within the QIIME2 pipeline (diversity plugin with the core-metrics-phylogenetic command) on rarefied data (2,503 contigs for 16S and 4191 contigs for ITS2).

### Prediction of microbial functions

To infer sequence based functional predictions aiming primarily at bacterial substrate digestion, the five treatment groups were analyzed using the standalone version of PICRUSt2 v2.5.3 [80] running the full pipeline with default settings (NSTI 2.0) on unrarefied data generated and filtered as described above within QIIME2. We restrict functional inference to a targeted and substrate-matched set of enzyme families that reflect dietary composition. Thus, the unstratified metagenome predictions were filtered based on EC or KO identifiers associated with maltase-glucoamylase and sucrase-isomaltase (dextrin degradation), alpha-amylase and maltogenic alpha-amylase (starch degradation), nitrogen fixation, endoglucanase and exoglucanases (cellulose degradation), pectinesterase and polygalacturonase (pectin degradation), endoxylanase, exoxylosidase, endomannanase, L-arabinosidase, endoarabanase and endogalactanase (hemicellulose degradation), lignin peroxidase, manganese peroxidase, versatile peroxidase, and laccases (lignin degradation) (Table S4). The results were subsequently normalized by calculating the z-score for each treatment and separately for the filtered EC and KO metagenome prediction files.

### Methane emission of BSFL and frass

To evaluate methane emissions from BSFL reared on industrial side streams and from the control larvae, from each feed treatment triplicates of pooled L5 larvae (1.5 g = 15–60 larvae) and the corresponding pooled frass sample (2 g) were collected from the boxes also used for larval growth curves measurement (see above). We used a pool of 10 adult cockroaches (*Shelfordella lateralis*) which are known to emit methane, as positive control [81]. Each sample was placed in a separate 120 ml glass vial sealed with a rubber stopper and incubated at 27 °C in the dark. Methane levels were then measured on an SRI 8610 gas chromatograph (SRI Instruments, Bad Honnef, Germany) equipped with a packed column (Porapak Q, 80/100 mesh, 274 cm by 3.18 mm inside diameter) and a flame ionization detector. Methane emissions were measured at three time points: at the starting point (t0), after two hours, and after four hours. Gas production rates were determined from the linear increase of gas concentration over time.

### Data analysis and statistics

Shapiro-Wilk test [82] was used to evaluate the normality of the nutritional composition of the feeds and larvae data and Brown-Forsythe [83] to test the homogeneity of variance in GraphPad Prism 10.2.1 (395) [84]. As the diet and larvae were the only experimental factors, the differences in nutritional composition within feeds and within larvae, respectively, were calculated in GraphPad Prism with one-way analysis of variance (ANOVA) followed by Tukey’s HSD post-hoc tests when data met parametric assumptions, as this approach is appropriate for comparing multiple groups with equal variances. If the normality or variance assumptions were not met, we applied Kruskal-Wallis test followed by Dunn’s post-hoc tests [85], as non-parametric tests provide a more robust alternative under these conditions. To evaluate relationships between nutritional composition (fat, protein, fiber, and starch) and larval performance (total larval developmental time, time to reach maximum weight, maximum larval weight), a Spearman rank correlation was conducted in GraphPad Prism [86]. Microbial community compositions were analyzed using rarefied data to calculate alpha and beta diversity, including species richness, Pielou’s evenness, Shannon index, and unweighted UniFrac distance matrix, all within QIIME2. Permutational multivariate analysis of variance (PERMANOVA) was conducted in R Statistical Software 4.3 [87] to compare microbial communities between groups (feed and gut) and treatments (CF, AP, PPI, PPL, RC) with R packages vegan 2.6–10 [88], and qiime2R 0.99 [89]. All figures presented were generated in R Statistical Software 4.4, along with the packages qiime2R 0.99, tidyverse 2.0.0 [90], ggh4x 0.3.0 [91], plyr 1.8.9 [92], ggpubr 0.6.0 [93], scales 1.3.0 [94], lubridate 1.9.4 [95], Dict 0.1.0 [96], and readxl 1.4.5 [97], as appropriate.

## Result

### Feed composition

We firstly analyzed the nutritional composition of the treatments, namely apple pomace (AP), potato pulp industrial-scale (PPI), potato pulp lab-scale (PPL), rapeseed cake (RC), and the control chicken feed (CF) to characterize differences in growth performance and associated gut microbial communities in BSFL reared on biogenic industrial side streams. The samples were collected and dry matter (DM) content determined, which ranged between 9.06 ± 0.60% for PPI and 94.07 ± 0.11% for RC (Table 1; Table S5). Samples were then lyophilized to exclude any remaining water. The measured crude ash content ranged from 1.24 ± 0.29% DM in AP to 12.94 ± 1.14% DM in CF, crude fiber content between 9.37 ± 3.30% DM in CF and 75.32 ± 5.20% DM in PPI, crude fat from 0.09 ± 0.11% DM in PPL to 17.16 ± 0.44% DM in RC, crude protein between 5.94 ± 0.94% DM in AP to 20.93 ± 0.20% DM in RC and starch from 0.23 ± 0.06% DM in RC to 62.34 ± 2.01% DM in PPL (Table 1; Table S5). The analyses revealed a high compositional heterogeneity in the feeds which presumably reflects differences in their nutritional value for BSFL.

**Table 1:** Compositional characterization of BSFL feeds including CF, AP, PPI, PPL, and RC, reported as percentage of dry matter (% DM), except for dry matter content (%). Data represent averages with standard deviations (*n* = 3). Distinct alphabetical letters (a–e) indicate significant differences between treatments for each analytical parameter (ANOVA, Tukey’s or Kruskal-Wallis, Dunn's, α = 0.05)

Feed	**CF**^**1**^	AP	PPI	**PPL**^**1**^	RC
Dry matter (%)	91.44 ± 0.04^b^	92.89 ± 0.51^a,b^	9.06 ± 0.60^d^	24.16 ± 1.20^c^	94.07 ± 0.11^a^
Crude ash (% DM)	12.94 ± 1.14^a^	1.24 ± 0.29^b^	5.88 ± 0.17^a,b^	5.93 ± 0.31^a,b^	6.26 ± 0.06^a,b^
Crude fiber (% DM)	9.37 ± 3.30^c^	34.27 ± 8.07^b^	75.32 ± 5.20^a^	10.75 ± 5.17^c^	12.44 ± 2.75^c^
Crude fat (% DM)	2.49 ± 0.04^a,b^	1.67 ± 0.14^a,b^	0.69 ± 0.08^a,b^	0.09 ± 0.11^b^	17.16 ± 0.44^a^
Crude protein (% DM)	18.24 ± 0.48^b^	5.94 ± 0.94^e^	9.41 ± 0.2^c^	7.61 ± 0.04^d^	20.93 ± 0.20^a^
Starch (% DM)	44.59 ± 1.41^b^	1.15 ± 0.65^d^	15.42 ± 0.6^c^	62.34 ± 2.01^a^	0.23 ± 0.06^d^

Data for CF and PPL have been published before except for starch [44] and are included for comparison

### BSFL growth and composition

**Fig. 1:**
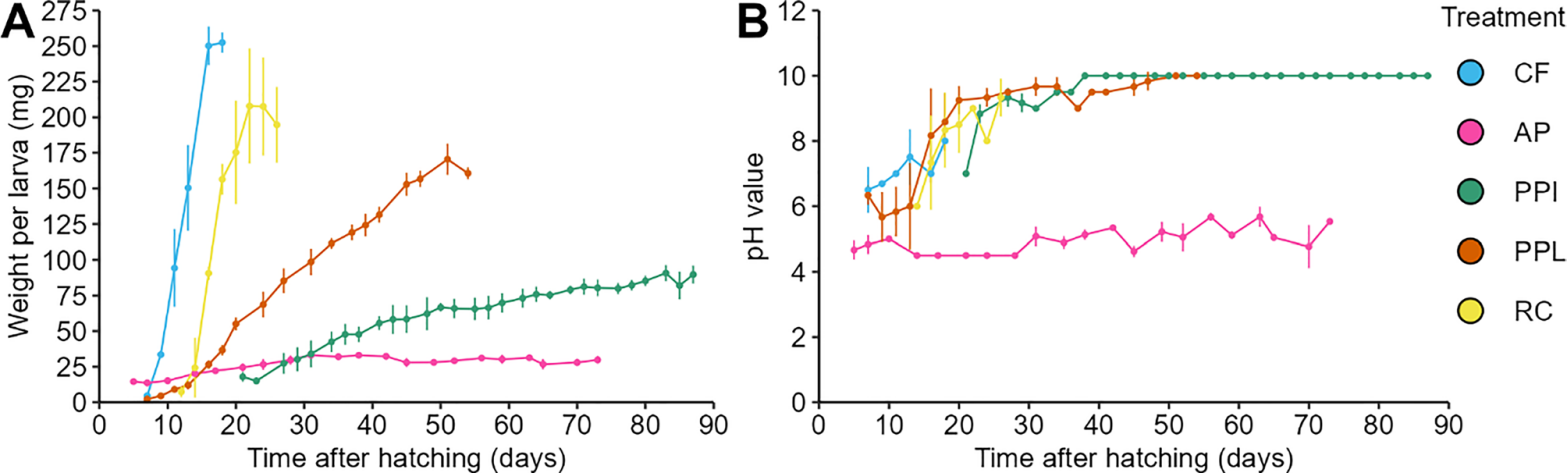
Larval growth performance and frass pH. (**A**) growth curves of BSFL reared on CF, AP, PPI, PPL, RC and (**B**) pH development during the experiment in the corresponding frass. The experiment was stopped when approximately ≥50% of larvae for each respective treatment reached prepupal stage. Error bars indicate standard deviation from three replicate boxes with *n* = 25 larvae per box for all treatments but PPI and PPL with *n* = 50. Growth curve data for PPL has been published before [44] and are included for comparison

Differences in the feed composition may affect BSFL growth and composition. Consequently, BSFL were reared on the above characterized feed treatments (CF, AP, PPL, PPI, and RC). Larvae reared on CF reached the prepupal stage after 17 days, followed by RC (25 days), PPL (52 days), AP (73 days), and PPI (87 days) (Fig. 1A). Importantly, all feeds supported larval growth and pupation despite the partially long developmental time, especially in AP and PPI. Maximal mean larval weight varied strongly between the feeds and ranged from 33.15 mg (AP) to 252.52 mg (CF) (Table 2). Prior to entering the prepupal stage, BSFL weight commonly decreases as they cease feeding, search for a suitable place and pupate (see CF, RC, and PPL in Fig. 1A). We further evaluated the associations between feed nutrients (crude fat, crude protein, crude fiber, and starch) and BSFL growth performance (Table S11). Crude fat was negatively correlated with time to reach maximum weight (ρ = −0.78; *p* = 0.001) and total larval developmental time (ρ = −0.59; *p* = 0.05), while showing non-significant correlation with maximum larval weight (ρ = 0.41; *p* = 0.12). These associations suggest that feeds with higher fat content are associated with shorter development times. Crude protein was also negatively correlated with time to reach maximum weight (ρ = −0.49; *p* = 0.07) and total developmental time (ρ = −0.59; *p* = 0.02), and positively correlated with maximum larval weight (ρ = 0.73; *p* = 0.003), indicating that higher crude protein levels lead to faster larval growth and increased biomass accumulation. In contrast, crude fiber was positively correlated with time to reach maximum weight (ρ = 0.64; *p* = 0.02) and total developmental time (ρ = 0.83; *p* = 0.001), but negatively correlated with maximum larval weight (ρ = −0.74; *p* = 0.01). Furthermore, the heterogenous growth performance of BSFL larvae reared on different feed types correlates with the compositional differences in the larvae, which likely reflects the nutritional value of the feed.

**Table 2:** BSFL growth on CF, AP, PPI, PPL, and RC. Values are reported as mean ± standard deviation from three replicate boxes with each 25 larvae (except PPI and PPL, *n* = 50) pooled

Feed	Maximum Larval Weight (mg)	Time to Reach Maximum Weight (days)
CF	252.52 ± 7.06	17
AP	33.15 ± 1.92	31
PPI	90.77 ± 5.66	83
PPL	170.47 ± 10.91	50
RC	207.87 ± 40.39	21

We further monitored the pH level in the frass that could indicate differences that directly or indirectly influence larvae growth performance (Fig. 1B). The initial pH in CF, PPL, PPI, and RC without BSFL ranged between 6.0 to 6.5. However, we observed an increasing pH value of the frass over time to a maximum of 8 (CF) to almost 10 (RC, PPL, PPI) during the experiment. There were no apparent differences of pH levels between these treatments. In contrast, pH in the AP frass was consistently lower ranging between 4.0 and 5.0. Interestingly, the low pH in AP frass correlated with the lowest tested growth performance for BSFL reared on this side stream.

To further characterize the effect of different feeds on BSFL growth, we assessed the larval composition by analyzing their crude ash, crude fat, and crude protein content. Larval crude ash content varied between samples and ranged from 4.85 ± 0.24% DM (CF) to 10.75 ± 0.67% DM (PPI) (Table 3; Table S6). Crude fat content was not significantly different between AP and PPI but differed significantly towards and between all other samples (CF, PPL, RC) and ranged from 4.01 ± 2.09% DM (PPI) to 33.94 ± 1.33% DM (CF). Crude protein content was lowest in CF with 40.68 ± 1.43% DM and highest in PPI with 60.08 ± 2.92% DM. Apart from the comparison of AP with RC and between AP with PPL, all other treatments differed significantly (Table 3; Table S6).

**Table 3:** Compositional characterization of BSFL reared on CF, AP, PPI, PPL, and RC, reported as percentage of dry matter (% DM). Data are depicted as averages with standard deviations (*n* = 3). Distinct alphabetical letters (a–d) indicate significant differences between treatments (ANOVA, Tukey’s or Kruskal-Wallis, Dunn's, α = 0.05)

Larvae	**CF**^**1**^	AP	PPI	**PPL**^**1**^	RC
Crude ash (%DM)	4.85 ± 0.24^c^	7.45 ± 0.47^b^	10.75 ± 0.67^a^	5.89 ± 0.33^c^	9.62 ± 0.48^a^
Crude fat (%DM)	33.94 ± 1.33^a^	7.17 ± 2.11^d^	4.01 ± 2.09^d^	16.83 ± 0.86^c^	25.11 ± 0.76^b^
Crude protein (%DM)	40.68 ± 1.43^d^	52.28 ± 0.54^b,c^	60.08 ± 2.92^a^	49.59 ± 0.43^c^	55.11 ± 0.59^b^

Data for CF and PPL larvae have been published before [44] and are included for comparison

Differences in developmental time, larval weight, and composition support our first hypothesis that rearing BSFL on compositionally different industrial side streams drives larval growth performance. Our analyses suggest that the combination of protein and fat in CF and RC favor larval growth, which appears to be further facilitated by an increased starch content in CF, resulting in CF and RC reared BSFL produced high biomass while developmental time was short compared to the other feeds (i.e., AP, PPI, and PPL) (Fig. 1; Tables 1, 2, 3).

### Bacterial and archaeal community analysis

Bacteria are known to be key players in insects’ digestion and health. To characterize the bacterial and archaeal gut microbiota of BSFL reared on different feed treatments as well as the bacterial and archaeal communities associated with each treatment type, we performed 16S rRNA gene amplicon sequencing on 60 samples (45 gut and 15 feed), yielding a total of 2,129,540 read pairs. After quality trimming, denoising, merging, chimera detection, and classification all three RC feed samples were excluded from further analysis based on 99% non-target amplifications. After removing 96 non-bacterial and non-archaeal ASVs from the dataset, the remaining 57 samples (45 gut and 12 feed) yielded a total of 1,156,687 contigs and 507 ASVs for further analysis. The number of contigs per sample ranged from 772 (AP-feed-02) to 33,633 (RC-gut-02) with an average of 22,293 (Fig. S1). Importantly, we did not identify any archaeal sequences in the dataset.

**Fig. 2:**
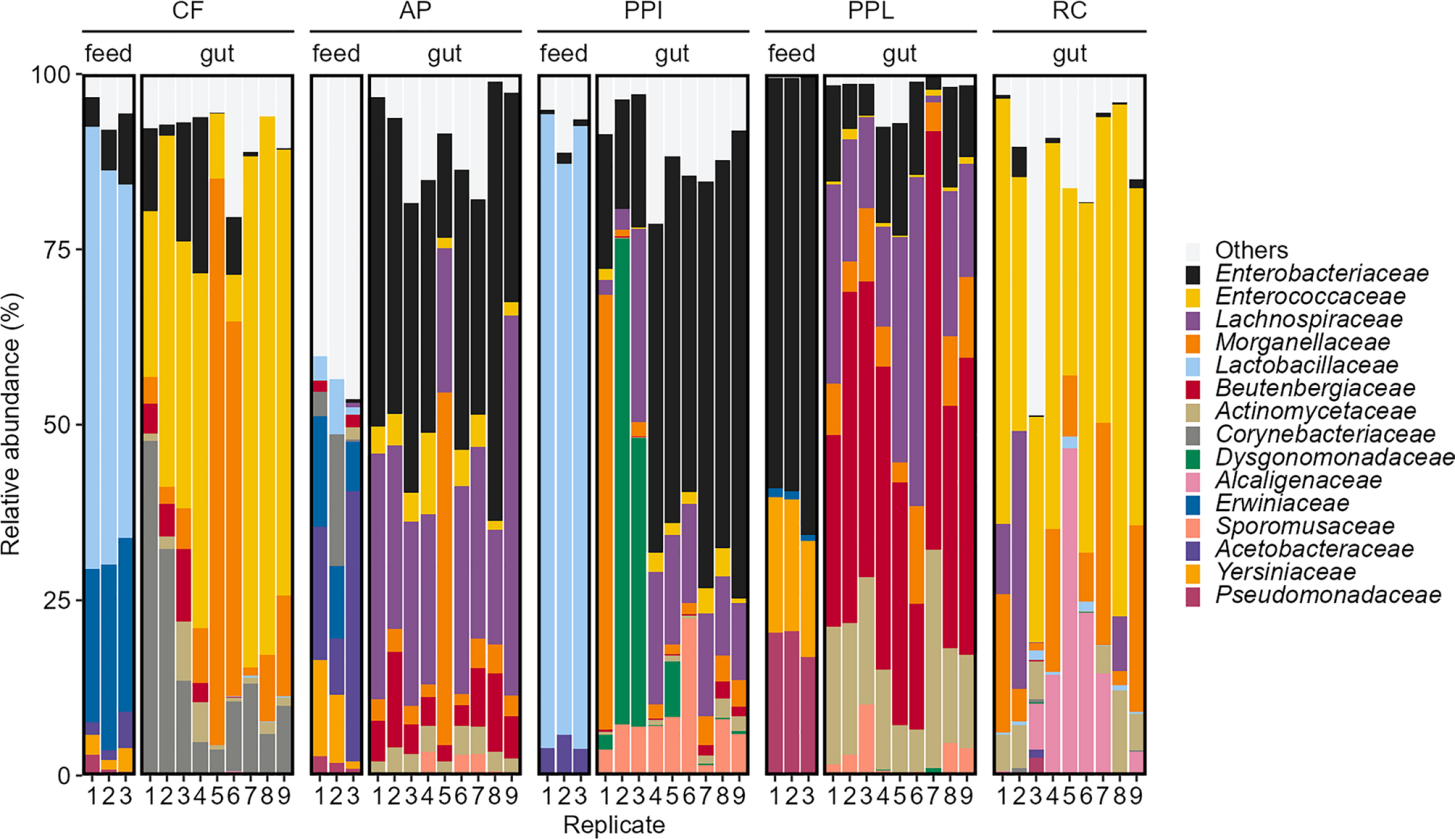
Bacterial community composition associated with insect feeds and BSFL guts. Stacked column plot of 16S rRNA gene amplicon data detailing relative bacterial abundances in CF, AP, PPI, PPL, and in guts of BSFL reared on CF, AP, PPI, PPL and RC. The 15 most abundant families across all samples are displayed, the remaining 68 families are grouped as “Others”. Relative abundances are based on unrarefied data

To characterize the BSFL gut and the feed associated bacterial communities, we then classified all sequences to the taxonomic levels of family (Fig. 2) and genus (Fig. S2). Gut samples differed distinctively from feed samples irrespective of the treatment feed. Between feed treatments (i.e., CF, AP, PPI, PPL, and RC), gut and feed samples both also differed from each other highlighting a treatment effect.

BSFL gut samples were generally dominated by *Enterobacteriaceae* (19.7% across all samples), *Enterococcaceae* (19.5%), and *Morganellaceae* (11.7%), together contributing 50.9% of all gut-associated bacterial ASVs. These families were present and relatively evenly distributed in all gut samples, though *Enterococcaceae* were particularly abundant in CF and RC guts while *Enterobacteriaceae* were most abundant in PPI and AP guts. *Beutenbergiaceae* were abundant in PPL gut (38.8%) and considerably less in CF, AP, PP, and RC gut (0–6.5%), whereas *Dysgonomonadaceae* were only present in PPI gut (13.5%). *Lachnospiraceae* were abundant in the guts of AP, PPI, PPL, RC (6.1–28.9%) and absent in CF gut. Based on the dominant families, there seem to be similarities between CF and RC as well as between AP and PPI gut samples. We further observed several taxa to be abundant in the gut samples that were rare or absent in the feed samples, such as *Beutenbergiaceae, Lachnospiraceae*, and *Dysgonomonadaceae.* Feed type samples were also heterogenous in their bacterial communities: *Lactobacillaceae* dominated CF and PPI feeds and were lacking in PPL. AP feed was dominated by *Acetobacteraceae* (21.8%) and PPL feed by *Enterobacteriaceae* (60.9%). *Lactobacillaceae* (49.2% across all samples, except PPL), *Enterobacteriaceae* (17.2%), and *Erwiniaceae* (12.2%, except PPI) contributed 78.6% of all feed-associated bacterial ASVs.

Reflecting the differences on the family level, the generic distribution was also heterogenous (Fig. S2). *Enterococcus* was particularly abundant in CF (43.5%) and RC gut (47.2%), whereas *Morganella* and *Providencia* showed varying relative abundances across all gut samples (0–74.3% and 0.3–35.3%, respectively). On the other hand, an unassigned genus of the *Enterobacteriaceae* family was the most prominent in PPL feed (60.5%) but was particularly abundant in AP gut (36.4%) and PPI gut (42%). *Lactobacillus* was abundant in PPI feed (59.1%) but was absent in all gut samples (Table S1).

To further detail differences between samples groups (i.e., gut and feed) and treatments (i.e., CF AP, PPI, PPL, and RC), we assessed alpha diversity measures from each sample. For statistical analyses, samples were rarefied to 2503 contigs per sample to avoid skewing of the data. AP-feed-01 and 02 were omitted for not meeting the cut-off. The average estimated species richness ranged from 22.67 ± 3.87 for PPL gut to 47 for AP feed. Average Pielou’s evenness ranged between 0.55 ± 0.09 for PPI gut and 0.78 for AP feed, while Shannon index was highest for AP feed with 4.34 and with 2.63 ± 0.32 lowest in PPL gut (Table 4).

**Table 4:** Alpha diversity indices calculated from rarefied 16S rRNA gene amplicon sequencing data from CF, AP, PPI, PPL, RC and the corresponding BSFL gut samples reared on these. Denoted are averages and standard deviation (SD).

Group	Treatment	*n*	Species Richness		Pielou’s evenness		Shannon index	
			Average	SD	Average	SD	Average	SD
Feed	CF	3	39.33	9.50	0.76	0.03	4.02	0.37
	AP	1	47	–	0.78	–	4.34	–
	PPI	3	45	5.57	0.64	0.03	3.49	0.28
	PPL	3	23.33	1.15	0.63	0.04	2.86	0.14
Gut	CF	9	40.78	8.56	0.60	0.15	3.21	0.88
	AP	9	33.22	5.74	0.64	0.09	3.24	0.58
	PPI	9	39.33	9.70	0.55	0.09	2.92	0.59
	PPL	9	22.67	3.87	0.59	0.06	2.63	0.32
	RC	9	45.33	17.04	0.61	0.10	3.29	0.67

**Fig. 3:**
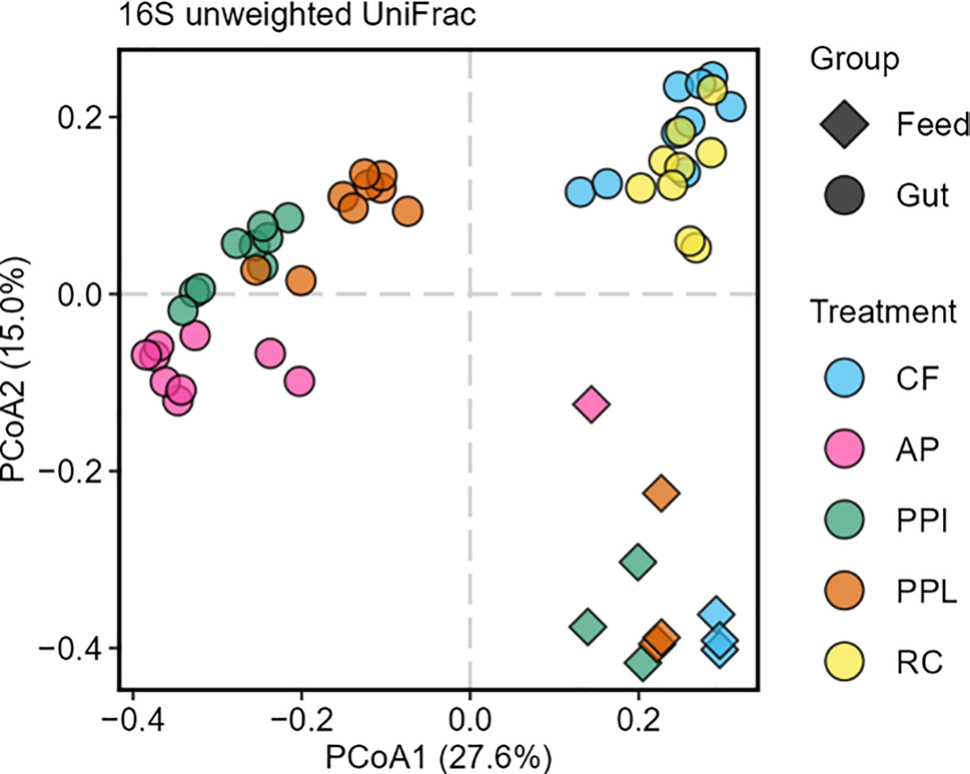
Visualization of differences in bacterial community composition from feed and BSFL gut samples. Principal coordinates analysis (PCoA) based on unweighted UniFrac detailing bacterial community structure in CF, AP, PPI, PPL, and the gut communities of BSFL reared on CF, AP, PPI, PPL and RC. Data are based on rarefied data

To visualize the differences in the bacterial communities in feed and gut samples, the data were plotted in an ordination plot (Fig. 3). Principal coordinate analysis (PCoA) of the unweighted UniFrac distance matrix showed a separate clustering of feed and gut samples. We performed two permutational multivariate analyses of variance (PERMANOVA), one test will analyze the differences between all feed and gut samples, while another will compare gut samples from high protein feeds (i.e., CF and RC) to those from low protein feeds (i.e., AP, PPI, and PPL).

The feed samples are clustering together and away from the gut samples (Fig. 3). Statistical testing of the differences between feed and gut samples were significant (PERMANOVA: R^2^ = 0.089, *p* = 0.001; Table S7), highlighting distinct bacterial community compositions between feed and gut. In contrast, gut samples from high protein feed treatments (CF, RC) clustered closely together and away from gut samples of low protein feed treatments (AP, PPI, PPL), which also clustered together (PERMANOVA: R^2^ = 0.353, *p* = 0.001; Table S7) (Fig. 3). Distinct bacterial gut communities that differ from their feed source and from each other depending on the feed type support our second hypothesis and suggest firstly, that the BSFL gut is a selective environment, and secondly, that the community is strongly influenced by the feed type.

### Prediction of microbial function and methanogenesis

To gain insights on the functional potential of BSFL gut bacteria, we used PICRUSt2 for sequence-based metabolic prediction. Our functional inference focused on enzyme families associated with the nutritional characteristics of each side stream. We particularly aimed for enzymatic pathways connected to cellulose degradation, dextrin degradation, hemicellulose (xylans, mannans, arabinans, galactans) degradation, nitrogen fixation, pectin degradation, and starch degradation (Table S4). All ASVs were below the maximum NSTI cutoff of 2.0 and were therefore retained for subsequent analyses.

**Fig. 4:**
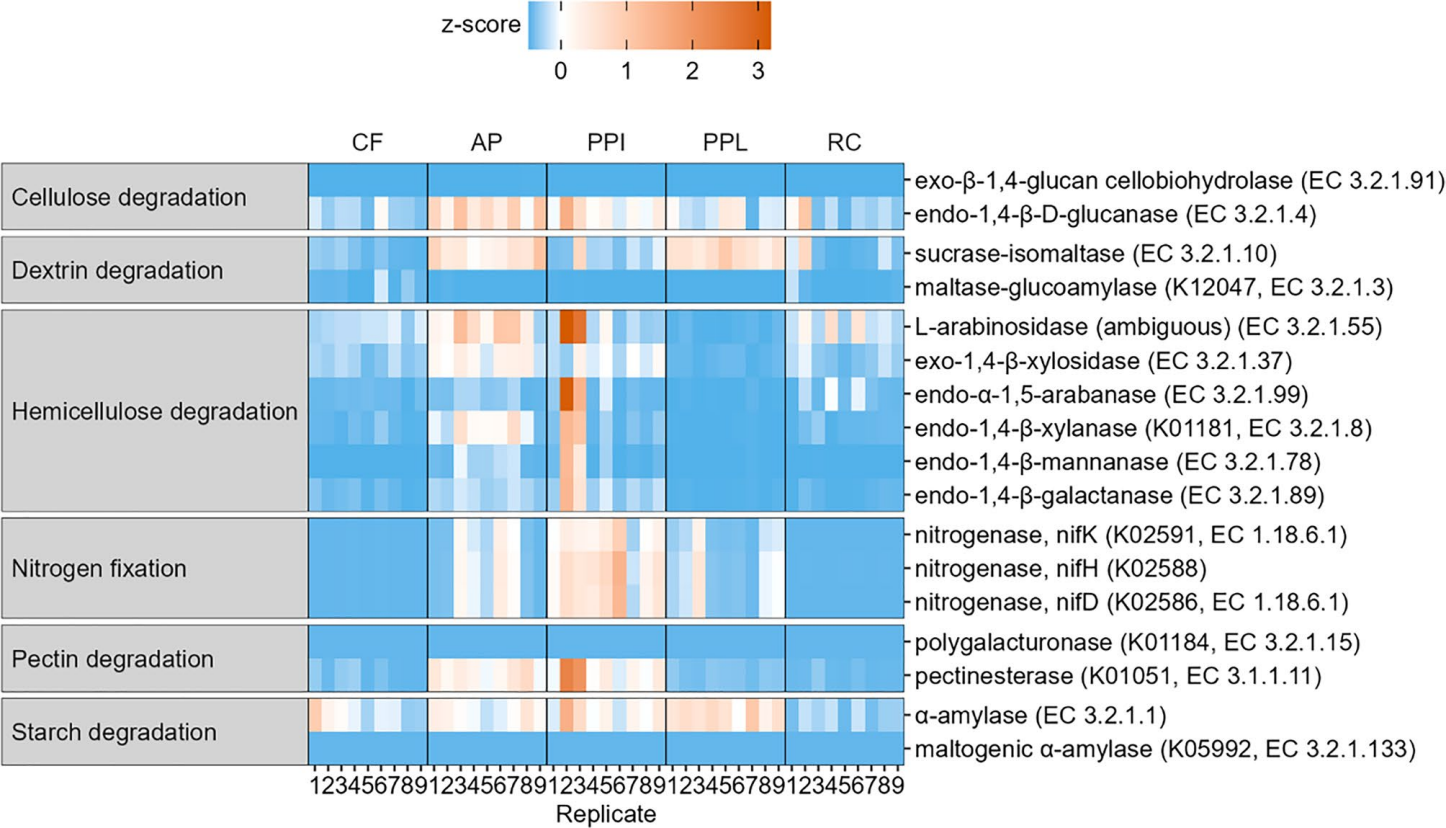
Heatmap of manually selected predicted functional pathways based on sequence data from CF, AP, PPI, PPL, and RC reared BSFL gut samples. The analyses are based on unrarefied data. The search pathway is denoted in parentheses

PICRUSt2 is apredictive tool that estimates the presence and abundance of functional genes based on the sequences of the 16S rRNA gene, using phylogenetic placement relative to sequenced reference genomes, and can therefore be used to infer the functional potential of bacterial communities [98]. Although this method is cost-effective for exploring potential functional differences across treatments, it is constrained by the availability of reference genomes and phylogenetic dependency. Consequently, its accuracy is reduced for poorly characterized or rare taxa. Furthermore, PICRUSt2 infers genomic potential rather than direct functional activity, and it cannot resolve strain-level functional variation [80]. Consequently, the results presented here represent predicted functional trends rather than direct measurements and should be interpreted as hypothesis-generating. We observed distinct patterns of putative function enrichment and depletion (Fig. 4). For example, genes for exo-β-1,4-glucan cellobiohydrolase, maltase-glucoamylase, polygalacturonase, and maltogenic α-amylase were depleted across all samples. In contrast, none of the considered pathways was enriched across all samples. Genes for endo-1,4-β-D-glucanase were enriched in AP and PPI guts. Genes for sucrase-isomaltase were enriched in AP and PPL guts. Genes for L-arabinosidase were partially enriched in AP and PPI guts. The genes involved in nitrogen fixation (nifK, nifH and nifD) were depleted in CF and RC guts, partially enriched in AP and PPL guts, but enriched in PPI guts. Genes for pectinesterase were enriched in AP and PPI guts. Genes for alpha-amylase were enriched in PPL and partially enriched in CF, AP, and PPI.

To functionally confirm the absence of methane producing archaeal taxa that were not detected in the 16S dataset, we tested for L5 BSFL- and frass-based methane production. In comparison to cockroach controls (emission rate of 102 nmol g^−1^h^−1^), we found no detectable methane concentration after four-hour incubations. This is supporting our third hypothesis indicating no or negligible methane production in the employed insect feed and BSFL treatments under tested laboratory conditions.

### Fungal community analysis

**Fig. 5:**
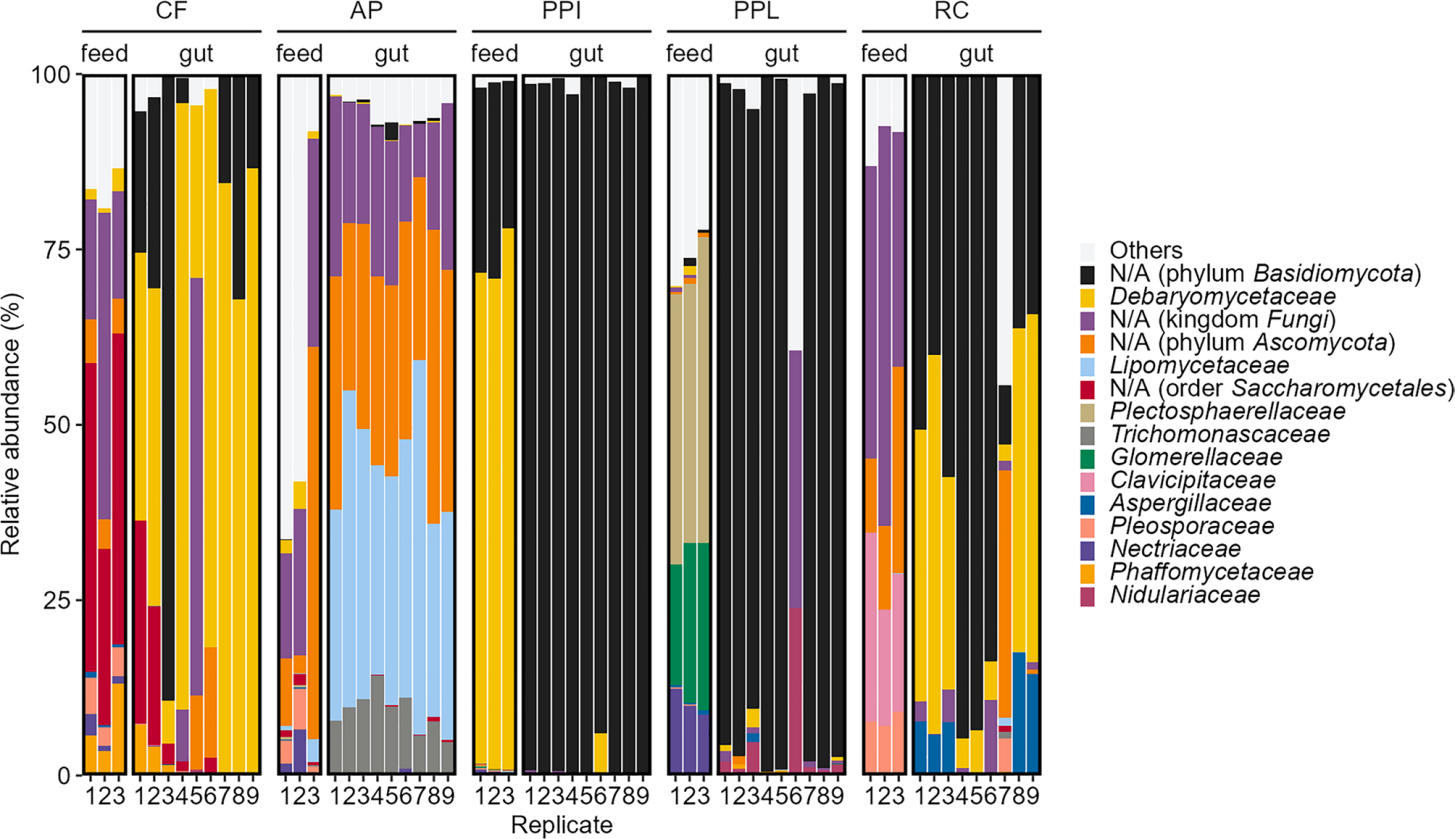
Fungal community composition. Stacked column plot of ITS2 amplicon sequencing data detailing fungal community composition associated to feeds and BSFL gut reared on these feeds, including CF, AP, PPI, PPL, and RC. The relative abundances of ASV are annotated to the family level. The 15 most abundant families across all samples are shown, the remaining 119 families are grouped as “Others”. The relative abundances are based on unrarefied data

To gain further insights on the microbial communities, we characterized the BSFL gut fungal communities from the same samples (gut and feed associated fungal communities, 5 different treatments) used for bacterial profiling. The targeted ITS2 region of the 60 samples (45 gut and 15 feed) were sequenced, resulting in a total of 1,650,452 read pairs. Only the forward reads were used for further analyses due to insufficient overlap of the corresponding reads. Following quality trimming, denoising, and chimera detection, we gained 1696 distinct amplicon sequence variants (ASVs) of which 1005 were removed as non-target amplifications (e.g., *Hermetia*, *Malus*, and *Solanum*). In total, 1,410,785 single-end reads were retained with 348 to 86,111 reads per sample (Fig. S1). All sequences were classified to the taxonomy level family (Fig. 5).

Gut and feed samples generally differed between each other and between different feed treatments. However, PPI, PPL and partially RC gut samples seemed similar based on the dominating unassigned family from the phylum Basidiomycota. This family generally dominated the gut samples (52.2%). The genus *Diutina* was found to be relatively abundant in the guts of larvae reared on CF (6.1–86.4%) and RC (0.2–54.1%), while AP guts were relatively abundant by genus *Dipodascopsis* (27.5–53.4%) and *Ascomycota*_*gen*_*Incertae*_*sedis* (23.8–41.9%) (Fig. S2).

Feed samples were also heterogenous and generally dominated by unassigned family from kingdom Fungi (0–57%). We observed particularly *Debaryomycetaceae* was abundant in PPI (69.9–77.1%). On the other hand, *Glomerellaceae* (17.2–23.8%) and *Plectosphaerellaceae* (36.9–43.7%) were abundant in PPL, while unassigned family from order Saccharomycetales was abundant in CF (24.9–44.1%). Furthermore, *Clavicipitaceae* was abundant in RC (16.6–26.9%).

To further detail differences between sample groups (i.e., gut and feed) and treatments (i.e., CF, AP, PPI, PPL, and RC) we next assessed alpha diversity indices from each sample. For statistical analyses, samples were rarefied to 4191 reads to avoid skewing of the data. Samples CF-gut-05, PPL-feed-03, PPL-gut-03 and 06, all RC-feed samples and RC-gut-07 were omitted for not meeting the cut-off. The average estimated species richness ranged from 5.78 ± 1.92 for PPI gut to 107.67 ± 54.08 for AP feed. Average Pielou’s evenness ranged between 0.12 ± 0.08 for PPI gut and 0.66 ± 0.03 for PPL feed, while Shannon index was highest in AP feed (4.00 ± 1.41) and lowest in PPI gut (0.27 ± 0.18) (Table 5).

**Table 5:** Alpha diversity indices calculated from rarefied ITS2 amplicon sequencing data from CF, AP, PPI, PPL, and RC and the corresponding gut samples from BSFL reared on these. Denoted are averages with standard deviation (SD).

Group	Treatment	*n*	Species Richness		Pielou’s evenness		Shannon index	
			Average	SD	Average	SD	Average	SD
Feed	CF	3	59.33	14.57	0.53	0.01	3.13	0.24
	AP	3	107.67	54.08	0.60	0.14	4.0	1.41
	PPI	3	28.33	5.77	0.29	0.02	1.42	0.17
	PPL	2	40	7.07	0.66	0.03	3.50	0.02
Gut	CF	8	14.88	12.77	0.40	0.11	1.44	0.76
	AP	9	21.33	3.12	0.53	0.03	2.34	0.18
	PPI	9	5.78	1.92	0.12	0.08	0.27	0.18
	PPL	7	10.57	2.76	0.26	0.05	0.85	0.11
	RC	8	8.75	2.05	0.53	0.10	1.63	0.24

**Fig. 6:**
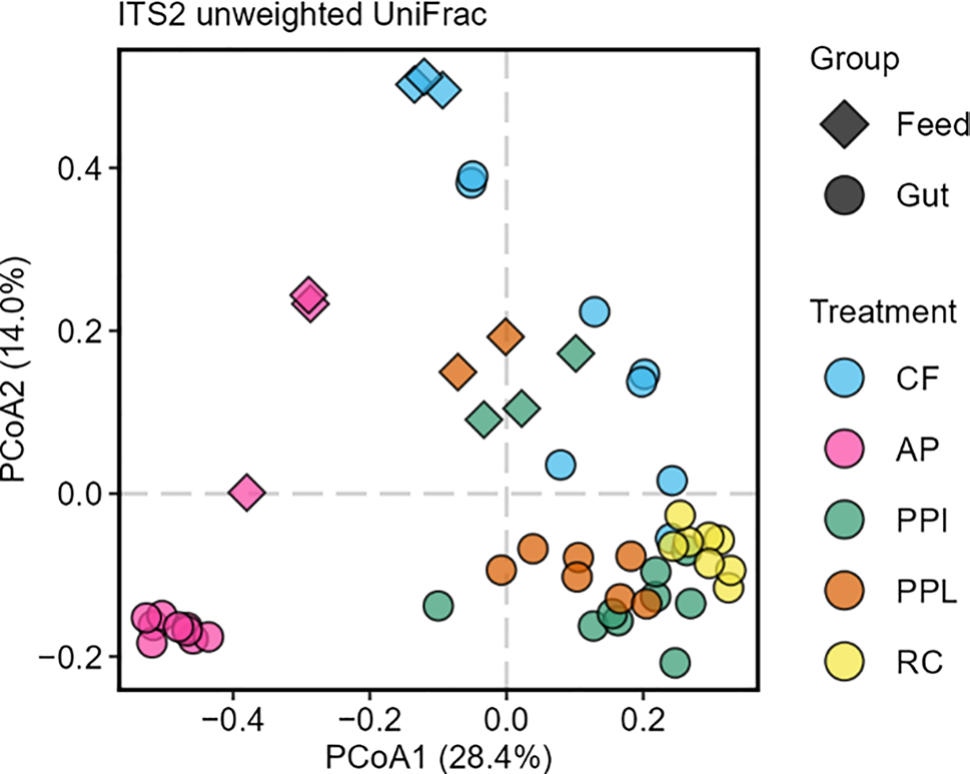
Visualization of differences in fungal community composition in feed and BSFL gut samples. PCoA based on unweighted UniFrac detailing fungal community structure in CF, AP, PPI, PPL, and the gut communities of BSFL reared on CF, AP, PPI, PPL and RC. Data shown based on rarefied data

To further compare the fungal communities between feed and gut samples, fungal communities were visualized in an ordination plot (Fig. 6). Principal coordinate analysis (PCoA) of unweighted UniFrac distance matrix showed that AP feed and gut samples clustered away from all other samples. To test for statistical differences, we performed two PERMANOVA analyses. First, we tested for differences between AP samples and all other samples. Second, we compared all feeds with all gut samples.

The gut samples PPI, PPL, and RC are clustered together, while particularly AP gut samples are clustered away from all other samples (PERMANOVA: R^2^ = 0.166, *p* = 0.001; Table S8). On the other hand, a separate clustering of feed and gut samples (PERMANOVA: R^2^ = 0.091, *p* = 0.001; Table S8), where all gut samples but CF clustered away from the feed samples. Similar to the previously described bacterial communities, the different feed types seemed to impact the fungal feed and the fungal gut communities.

## Discussion

Unlike many previous studies on BSFL that are primarily descriptive (e.g. ‘diet shifts taxa’), our work establishes clear links between real, regionally available side streams and (i) diet-nutritional profile-performance relationships; (ii) cross-kingdom (bacteria and fungi) gut community patterns; and (iii) consistent null results for methanogenesis as an ecologically relevant benefit. In addition, we explicitly explored substrate-specific functional signals in a targeted yet exploratory manner. Together, these approaches provide a hypothesis-driven, resource-efficient framework to inform the scaling up of insect production and the sustainable use of organic side streams.

Our data showed that the developmental time, larval weight and larval composition is strongly dependent on the feed chemistry. We further found the bacterial and fungal communities in the BSFL gut to differ remarkably depending on the side streams used as feed. Functional predictions further indicated that microbial metabolic potential varied across BSFL fed different side streams, reflecting differences in nutrient availability. Furthermore, the absence of methane emissions of BSFL and methanogenic archaea in guts of BSFL reared on these side streams indicates further advantages in terms of sustainability.

### Nutritional composition and larval growth

For a circular economy and resource-efficient, climate-friendly insect farming, low-cost, locally produced industrial side streams play a central role as feed sources. In this study, we used different plant-based side streams to investigate their suitability for BSFL farming by assessing growth performance and the associated microbial communities including their putative functional potential. The four tested industrial side streams, including AP, PPI, PPL and RC differed in their effects on BSFL growth performance and the associated gut microbial communities.

From a producer’s perspective, factors such as growth, yield, and production efficiency are key to profitability [99]. To evaluate the suitability of different industrial side streams for BSFL rearing, we tested AP, PPI, PPL, and RC. All of the larvae reached the prepupal stage on these substrates (Fig. 1A), indicating that they can support larval development in general. However, growth performance varied among the substrates, with some supporting faster development and higher larval weights than others. Our result showed that larvae fed with AP, PPI, and PPL reached the prepupal stage slower and gained less weight than the CF reference group (Fig. 1A, Table 2). The three side streams contained less fat and protein compared to CF and RC. High protein diets support BSFL weight gain and reduce developmental time [100–102]. Accordingly, in our experiments RC with 20.93% DM protein supported fast BSFL growth (25 d to reach the prepupal stage) and larval weight was only slightly lower (~21%) compared to the CF control.

In contrast, low protein likely contributes to reduced larval weight and prolongs the growth period [31]. Further, starch-rich and fat-poor diets may lead to enhanced fat content in BSFL [103]. This is in agreement with our observation that larvae reared on starch-rich PPL had 5-fold higher biomass and 4-fold higher fat content when compared to larvae reared on AP and PPI, respectively. Moreover, the difference in developmental time between the two potato-derived substrates is best explained by processing-driven changes in feed composition and accessibility, rather than processing per se. PPI is more extensively processed, which reduces nutrient accessibility and potentially alters the initial microbial community. In contrast, PPL was produced via mechanical juice extraction, which allows it to retain a more accessible nutrient fraction (starch; see Table 1). These differences in physical properties and nutrient availability likely shaped larval digestive and microbial dynamics. Overall, the protein and fat content of the industrial side streams (except RC) are quite low in comparison with CF (Table 1).

In addition to protein and fat, we also considered the fiber content of the feeds. We found a high amount of fiber in AP and PPI feed (Table 1) which possibly contributed to the slower development of the larvae, concomitant to the low protein and fat content (Table 2, Fig. 1A). This is also reflected in previous studies of BSF larvae. Broeckx et al. [34] reported that apple pulp, high in crude fiber (25.7%) and lignin (14.3%) while low in protein (3.4%), supported only low maximum mean larval weight of 38.3 mg. Similarly, BSFL fed on rice straw reached a maximum weight of 13.6 mg [104]. Rice straw is low in crude protein (3–6%) [105] and high in crude fiber (cellulose: 32.5%, hemicellulose: 19.8%, lignin: 6.5%) [106], thus not suitable as animal feeds. Klüber et al. [44] showed that the final larval weight for BSFL reared on lignocellulosic empty fruit bunches from the oil palm was 2.4-fold less than the larvae reared on CF and the larvae further required ~169 days to reach the prepupal stage. Overall, low protein and fat content combined with high fiber do not support an efficient BSFL development and a high biomass as indicated by the correlation analysis.

Another factor known to affect larval growth is the pH of the substrate. BSFL reared on diets with pH 2–4 showed a longer developmental time and a significantly lower weight in comparison to BSFL reared on diets with pH 6–10 [107]. In our study, we observed that the pH in AP feed and AP frass was consistently low, which could be contributing to slower the larval growth and development in AP and needs to be addressed in the future.

One promising strategy for improving side streams is to formulate or blend industrial side streams. Recent studies have demonstrated that blending coproducts or biowastes can significantly boost BSFL growth and bioconversion efficiency by balancing macronutrient ratios, diluting inhibitory compounds, and enhancing moisture and physical properties. Formulated or mixed substrates have produced better results than single feedstocks in both controlled trials and applied applications [108–111]. These findings suggest that targeted blending, particularly of acidic or fiber-rich feeds such as AP, is a practical next step for enhancing larval development and overall production efficiency, although our individual side streams data are important for understanding substrate-specific performance and microbiota dynamics.

### Insect feeds affect larval composition

In accordance with the differences in development described above, it is well established that insect feed drives the nutritional compositions of BSFL [33, 48, 112–114]. This particularly holds true in regard to the lipid content and composition [101], but also to the overall protein fraction [108, 115]. This offers adjustability for the application as animal feeds, where recent legal changes in the EU have promoted the use as feed for fish, poultry, and pigs [12–18]. For example, BSFL reared on mill by-products (3.0% fat, 14.5% protein, 51.7% fiber) yielded high protein larvae (42.1%) [109]. Feed with high protein content was found to promote high fat content and body mass of the larvae [116]. In line with the literature, we found a low crude fat content (4.0–7.1%) in the larvae reared on the high-fiber and low-protein feeds AP and PPI compared to the CF and RC larvae (25.0–33.9%). At the same time, the crude protein content of BSFL reared on AP and PPI was ~46% higher than that reared on CF and RC.

Carbohydrate- and therefore energy-rich diets can also contribute to elevated fat levels in the larvae. Here, for example amylases from the host contribute to starch degradation [117]. Carbohydrates are thus considered central for effective BSFL growth [118]. This corroborates our result that BSFL reared on starch-rich PPL yielded higher weights than those grown on AP and PPI. Importantly and as expected, the composition of the here tested side streams does not only affect larval development and growth, but also its composition.

Understanding the nutritional composition of black soldier fly larvae is essential for their effective inclusion in animal feed, as it determines their suitability for different animals. Factors such as protein, fat, and micronutrient content influence growth performance, feed conversion, and overall animal health. The selection of an ideal feed therefore also depends on the intended application of the larvae. For instance, a high-protein and low-fat composition for the larvae is preferred in aquaculture, while high-protein and fat larvae might be preferrable for poultry and certain pig production stages (e.g. grower/finisher) [119–122]. The here tested side streams offer a range of feeds that may be employed to optimize product composition and feed performance. In addition, insect-derived processed animal proteins in the EU are subject to specific processing and labeling requirements, and they are commonly commercialized in defatted or partially defatted forms for certain applications [123].

### Insect feeds impact BSFL gut microbiota

Many insects are known to support gut-associated microbes, which is largely attributed to the hosts ability to maintain a selected microbial community [36]. We restricted our analyses and interpretations to the family and, in some cases, the genus level. Our data confirm that feed and gut-associated microbial communities differ [33, 35] and therefore suggest that feed compositional differences drive the microbial communities. This is based on the significant differences between gut microbial communities of BSFL reared on different feed types (Tables 4 and 5; Figs. 3, 5, and 6). The differences may at least in part present functional optimized communities that support the breakdown and digestion of specific feed components [33, 35]. On the other hand, BSFL guts are also known to host several taxa, which are present regardless of diet, referred to as the core microbiome, that were also present in our samples [33, 35, 112, 114]. These include *Enterococcaceae*, *Enterobacteriaceae*, *Morganellaceae*, and *Actinomycetaceae* which were consistently present in all gut samples (Fig. 2).

The composition of the gut microbiota is thought to reflect functional adjustments, for example to support digestion. Many of the plant-based agricultural side streams consist of complex polymers (e.g., pectin, lignin, hemicellulose, cellulose, or starch). Cellulose degradation involves multiple CAZymes, that is endoglucanases, exoglucanases, and β-glucosidases, generally produced by bacteria [41]. Most animals have a limited number of enzymes for cellulose degradation. Microbial symbionts in the gut may enable their hydrolyses into monomers, which can be absorbed by the insect [36]. Certain bacterial families such as *Lachnospiraceae* appeared to be associated with BSFL fed with fiber-rich side streams AP and PPI. *Lachnospiraceae* are obligate anaerobic gut-associated bacteria [124, 125] that have the ability to metabolize fiber-rich, low-quality feedstocks [126]. On the other hand, BSFL fed with the high protein diets including CF and RC were preferentially associated with the genera *Enterococcus* and *Providencia*. Studies showed that *Enterococcus* was related to lipase activity which related to lipid conversion and possibly influencing host hunger and feeding behavior [127, 128], whereas *Providencia* suggested to be involved in the metabolism of protein and urea hydrolysis in BSFL gut [42, 129, 130]. The family *Dysgonomonadaceae* was only observed in PPL gut samples. Bruno et al. [38] emphasize the role of *Dysgonomonadaceae* in polysaccharide degradation (starch, pectin, cellulose), and nitrogen fixation [131, 132], which corroborates with our nutritional analysis that showed high starch and low protein (low nitrogen) content in PPL.

Analogous to the bacterial members, the fungal gut community was influenced by the feed type. *Debaryomycetaceae* was found to be relatively abundant in the gut of larvae reared on protein-rich feed (CF, RC), while Saccharomycetales were enriched particularly in AP guts. A high relative abundance of the genus *Diutina* was previously also observed in CF and protein-rich cottonseed press cake [33]. Further, unassigned families from the phylum Basidiomycota were highly abundant in PPI and PPL gut samples. Basidiomycota are common members in the potato rhizosphere microbiome [133] and were also detected in our PPI and PPL feed samples.

These findings confirm that the nutritional composition of the feed influences the fungal and bacterial communities in BSFL gut. Although the observed microbiota plasticity may partly reflect the presence of substrate-associated microbiota in the gut, the absence of certain taxa (i.e. *Beutenbergiaceae*, *Lachnospiraceae*, *Dysgonomonadaceae*) in the substrates suggests that the actual gut-associated microbial community is well represented. Conversely, the presence of several relatively highly abundant taxa, including *Lactobacillaceae, Yersiniaceae,* and *Erwiniaceae*, only in the substrates (Fig. 2, 5, S2 and S3; Table S1, S2) further supports this finding and indicates host selection. Furthermore, PCoA of 16S (Fig. 3) and ITS2 (Fig. 6) unweighted UniFrac distances support this distinction, showing clear clustering of feed samples away from gut samples. Statistical testing confirmed these differences, revealing significant separation between feed and gut communities for both bacteria and fungi. Overall, these results suggest that the gut microbiota is not merely a reflection of substrate-associated microbes, but rather a distinct community that is selectively maintained within the gut. The observed differences in microbiota between feed and gut likely arise from selection pressures in the gut, such as physicochemical conditions (e.g., pH and oxygen levels), enzymes, immune responses, or nutrient availability [36].

To explore functional adjustments of the BSFL gut microbiota, we employed sequence based functional predictions for the bacterial communities. We used PICRUSt2 as an exploratory tool to gain preliminary insights into functional trends in the gut microbiota, as has also been reported in studies of the gut microbiotas of other insects [134–137]. Our data on selected enzymatic pathways showed a heterogenous pattern that seems to reflect the feed nutritional composition (Fig. 4). Importantly, in most cases the feed composition seemed to largely support trends in putative functional enrichment and depletion patterns. For example, the gut microbiota of BSFL reared on fiber-rich and nitrogen-poor diets (AP, PPI) showed an enrichment of predicted enzymatic pathways involved in cellulose degradation (endo-1,4-β-D-glucanase), hemicellulose degradation (L-arabinosidase), pectin degradation (pectinesterase) and nitrogen fixation (nifK, nifH, nifD). Further, dextrin degradation (sucrose-isomaltase) was enriched in AP and PPL, while alpha amylase was enriched in all gut samples, except RC. However, no prediction for lignin-degrading enzymes (such as laccase and peroxidase) was found using PICRUSt2. Lignin degradation is mostly attributed to fungi, thus BSFL gut bacteria may not be able to completely degrade substrates with a high lignocellulose content [44]. Klüber et al. [35] demonstrated that pre-fermentation of a lignocellulose-rich substrate with filamentous fungi could improve the larval growth.

To date, studies on the BSFL gut microbiota have mostly used bacterial 16S rRNA gene amplicon sequencing to investigate its dynamics [48]. While the approach allows for a cost-effective and efficient profiling of entire bacterial communities, it has inherent limitations [138]. Closely related bacterial species often share highly similar 16S rRNA sequences, thereby rendering species-level identification unreliable [139]. Furthermore, as this method only targets a single taxonomic marker gene, it does not directly measure the microbial community’s functional capabilities [140].

The predicted functional profiles inferred using PICRUSt2 offer insight into the potential metabolic capabilities of gut-associated bacterial communities across tested substrates. However, these results should be interpreted within the context of the methodological limitations of the approach. PICRUSt2 uses phylogenetic inference and available reference genomes to estimate gene content from 16S rRNA data. This introduces reference genome bias, limiting prediction accuracy for poorly characterized or rare taxa. Importantly, PICRUSt2 infers genomic potential rather than direct functional activity; therefore, it does not capture gene expression or metabolic regulation. Consequently, observed differences in predicted pathways should be viewed as patterns that generate hypotheses about possible functional associations between diet, microbiota composition, and larval performance. Confirming these functional relationships will require direct methods, such as shotgun metagenomic or metatranscriptomic sequencing [98, 141, 142]. Therefore, functional predictions based on 16S data, such as those generated by PICRUSt2, should be considered exploratory and interpreted with caution. Nevertheless, the trends observed in this study appear to reflect functional optimization of the BSFL gut microbiota in response to different feeds. Metagenomic shotgun sequencing is a relatively recent approach in BSFL research, with only a limited number of studies having been published to date [143, 144]. Nevertheless, applying this method would enable species-level resolution and a more comprehensive characterization of the metabolic potential of BSFL-associated microbiota.

### Economic and ecological aspects

Availability and economic considerations of utilizing industrial side streams as feed for BSFL are crucial factors in scaling up productions. Repurposing abundant, low-cost by-products, BSFL rearing can reduce operational costs while simultaneously addressing waste management challenges. Primarily, insect feeds must provide support of sufficient biomass production and animal health. From a nutritional perspective, RC is a promising industrial side stream for optimizing the growth performance of BSFL, as our data demonstrate a short developmental time (25 days) and a high larval weight (207.8 mg) comparable to the larvae reared with conventional CF. Secondarily, insect feeds must be available at the required amounts and a competitive price. In Germany, approximately 250,000 tons of apple pomace and ~300,000 tons of rapeseed cake are produced annually as by-products of fruit processing and oil extraction. Over 1 million tons of potato pulp are obtained from starch manufacturing per year in Europe [145–147]. Therefore, the side streams are available at sufficient amounts to support considerable insect farming upscaling efforts.

To characterize the economical perspective of the side products, we also assessed the feed costs. The here tested side streams are currently used as livestock feed or raw materials for pectin extraction and microbial fermentation for compound production. The market prices range from €0 to €73.3/ton for apple pomace, €10/ton for potato pulp (Denmark), and €40 to €60/ton for rapeseed cake [28, 148, 149], whereas the cost of chicken feed used in this study is €877.6/ton. However, it is important to emphasize that the use of chicken feed was limited to controlled laboratory conditions for benchmarking and does not suggest its (economic) viability as a substrate in industrial applications.

Although rapeseed cake is widely used in conventional livestock rations, we evaluate its suitability for BSFL in a regional setting where supply is abundant and cost-competitive. We do not propose diverting material from existing livestock uses. Instead, its local availability, affordability, and the strong BSFL performance observed in our trials justify including rapeseed cake as a relevant feed ingredient alongside other side streams.

While low-cost and abundant side streams offer sustainability advantages, they may require additional processing or supplementation to match the protein productivity of conventional feeds. However, the possibility to scale-up the production of BSFL using industrial side streams also depends on seasonal availability, as they are linked to seasonal harvesting and processing periods. One effective strategy is to establish strong collaborations with agricultural and food processing industries, enabling timely collection around peak production periods. To preserve surplus materials, freezing offers a practical solution, particularly for high-moisture substrates like apple pomace and potato pulp, as it maintains nutritional quality and microbial integrity over extended periods [150]. However, related energy and storage costs would need to be factored in. Additionally, diversifying the types of side streams used, by incorporating other readily available agricultural residues, can reduce dependency on any single feedstock and mitigate supply fluctuations.

Adding to the sustainability of insect farming are considerations about climate gases. Insect farming is often promoted as a local option for protein production and waste management, thereby saving energy and CO_2_ emissions during transport [151, 152]. During animal protein production the GHG production is another considerable sustainability aspect [153]. Particularly traditional livestock feed sources, such as soybean meal, contribute to significant methane emissions due to enteric fermentation and waste decomposition [154]. Previous studies have shown the potential of BSFL to produce methane during bioconversion activities on certain substrates, such as food waste [55], wheat bran and flour [155], orange peels and food waste [156]. Methanogens are a diverse group of archaea that produce methane and are found in various anoxic habitats, including the digestive tracts of insects [157], where they produce methane as a by-product from energy production. Methane is a potent greenhouse gas with a global warming potential 28–38 times that of carbon dioxide (CO₂) over a 100-year timeframe [158]. The economic implications of methane emissions are crucial considering carbon pricing mechanisms.

In Germany, the carbon price is projected to reach €55 per ton CO₂-equivalent by 2025 [159], which translates to an approximate cost of €1,540 per ton of methane emitted. For example, one single cow produces around 70–120 kg of methane [160] with additional costs of €3.85–6.6 per ton of methane. In contrast, our study shows that no archaeal 16S rRNA gene reads were found in the gut community data of L5 BSFL reared on the tested industrial side streams and we did not detect any methane emission of L5 BSFL in vial assays, while positive controls produced clear methane signals. These results indicate that methanogenesis was not detectable in actively feeding L5 BSFL under our laboratory conditions. Nevertheless, as larval metabolism changes across instars, comprehensive measurements from early larval stages through to adult BSF are required, alongside archaeal *mcrA*-targeted assays or metagenomic approaches, to confirm the absence of methanogens in BSFL. While the costs for climate gases in agricultural production are currently either hidden or not factored into the products [153], they present a quantifiable advantage of employing the here tested biogenic side streams and insect protein production further highlighting options for sustainable animal protein production.

Our study demonstrates that regionally available, low-cost side streams can support BSFL growth and offer preliminary sustainability advantages. However, a full comparative cost–benefit or life cycle assessment relative to conventional protein sources was beyond the scope of this work. Future studies that incorporate large-scale rearing data, energy inputs, transportation costs, and processing costs are needed to rigorously quantify the economic and environmental implications of producing BSFL on these side streams. Additionally, while we monitored substrate pH as an environmental parameter, other factors such as temperature, humidity, and larval density were not continuously measured. Their interactions with feed composition may affect growth outcomes. Incorporating continuous monitoring of environmental conditions in future studies will clarify how these factors, combined with diet, shape larval performance and microbial community dynamics in industry-relevant settings.

## Conclusion and outlook

Here, we have demonstrated that the tested low-cost industrial side streams (AP, PPI, PPL, RC) can support BSFL development under the conditions of our study. RC has proven to produce the best performance on BSFL growth and developmental time among all tested side streams. Not surprisingly, high protein content supports efficient growth, as shown by higher larval weight and shorter developmental time. In contrast, primarily the combination of low protein and fat content, in addition to the high fiber content in the feed detrimentally negatively affected the larval growth performance. While CF supported the fastest growth and highest maximal weight for all feeds, the availability and costs for the tested side streams and particularly RC appear attractive. Furthermore, our measurements of BSFL reared on the tested side streams under laboratory conditions did not detect methane. However, this was based on a single measurement, and methane emissions were not the primary focus of this study. Therefore, the result should be interpreted cautiously. Our findings highlight how the substrate not only affects larval development but also shapes the structure and functional potential of the gut microbial communities. The functional predictions provided by PICRUSt2 offer preliminary insights into the microbial metabolic potential. However, there is a need for comprehensive metagenomic sequencing and expression profiling to confirm the mechanistic links between the microbiota, feed chemistry, and larval performance. The observed microbial shifts suggest that specific taxa may contribute to the degradation of complex dietary components and support nutrient absorption.

Future work should assess how the side streams can be optimized, for example by improved feed formulations and processing. Mixing different industrial side streams offers a promising approach to enhance the nutritional profile of the feed while maintaining economic feasibility. Additionally, employing cultured microbial members could be optimized to support pre-digestive external fermentation of the feed as well as the establishment of efficient, side stream optimized gut microbial communities potentially facilitating the feed conversion internally.

## Electronic supplementary material

Below is the link to the electronic supplementary material.

Supplementary Material 1

Supplementary Material 2

Supplementary Material 3

Supplementary Material 4

Supplementary Material 5

Supplementary Material 6

Supplementary Material 7

Supplementary Material 8

Supplementary Material 9

Supplementary Material 10

## Data Availability

Raw sequencing reads are available under the Project PRJEB87652 at the European Nucleotide Archive (ENA, https://www.ebi.ac.uk/ena/).
