# Supplementary material for: Diet chemistry and cross-kingdom microbiota associate with black soldier fly larvae performance on regional side streams
Source: Anim Microbiome. 2026 Jan 8;8:20. doi: 10.1186/s42523-025-00509-6 (PMC12908405; doi:10.1186/s42523-025-00509-6)
Supplement: Supplementary file 1 — Supplementary Material 1 [file 42523_2025_509_MOESM1_ESM.docx]

## Supplementary data


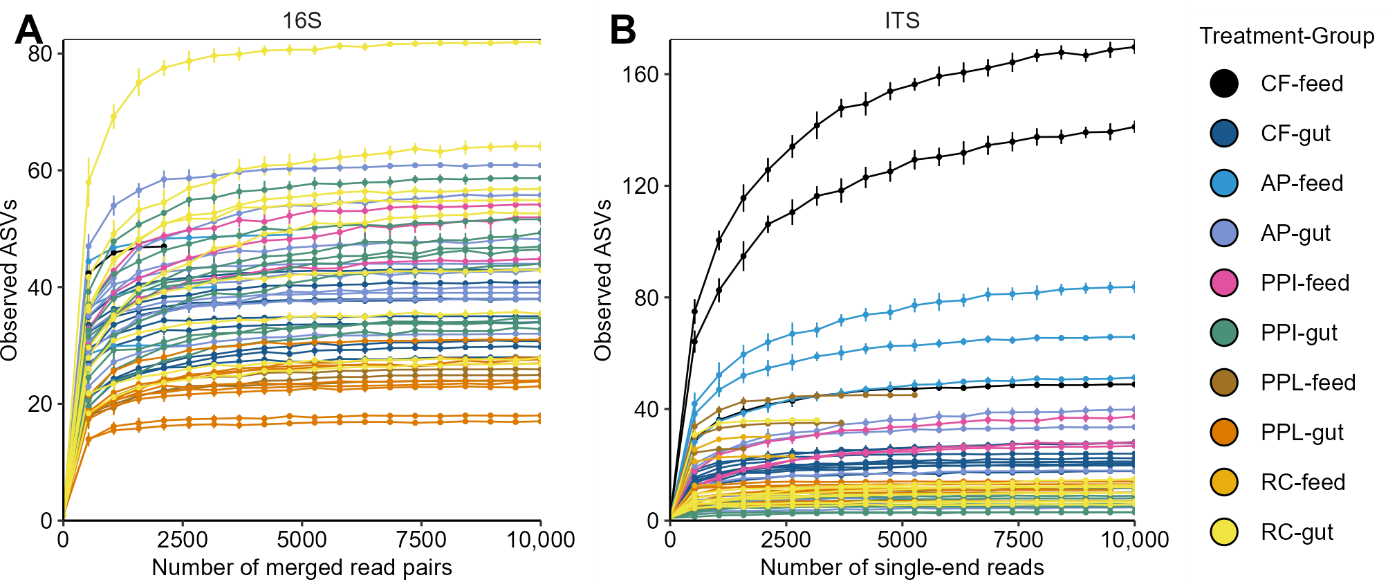


Figure S1. Alpha rarefaction curves detailing the sequencing depth for (**A**) 16S rRNA and (**B**) ITS2 amplicon sequencing after removing unwanted sequences (mitochondrial, chloroplast, unclassified, or wrong kingdom). Data based on the number of observed amplicon sequence variants (ASV). Mean values from 20 iterations per step with calculated standard deviation from gut samples and from five treatments chicken feed (CF), apple pomace (AP), potato pulp industrial-scale (PPI), potato pulp lab-scale (PPL), and rapeseed cake (RC) are shown. Replicates share the same color.


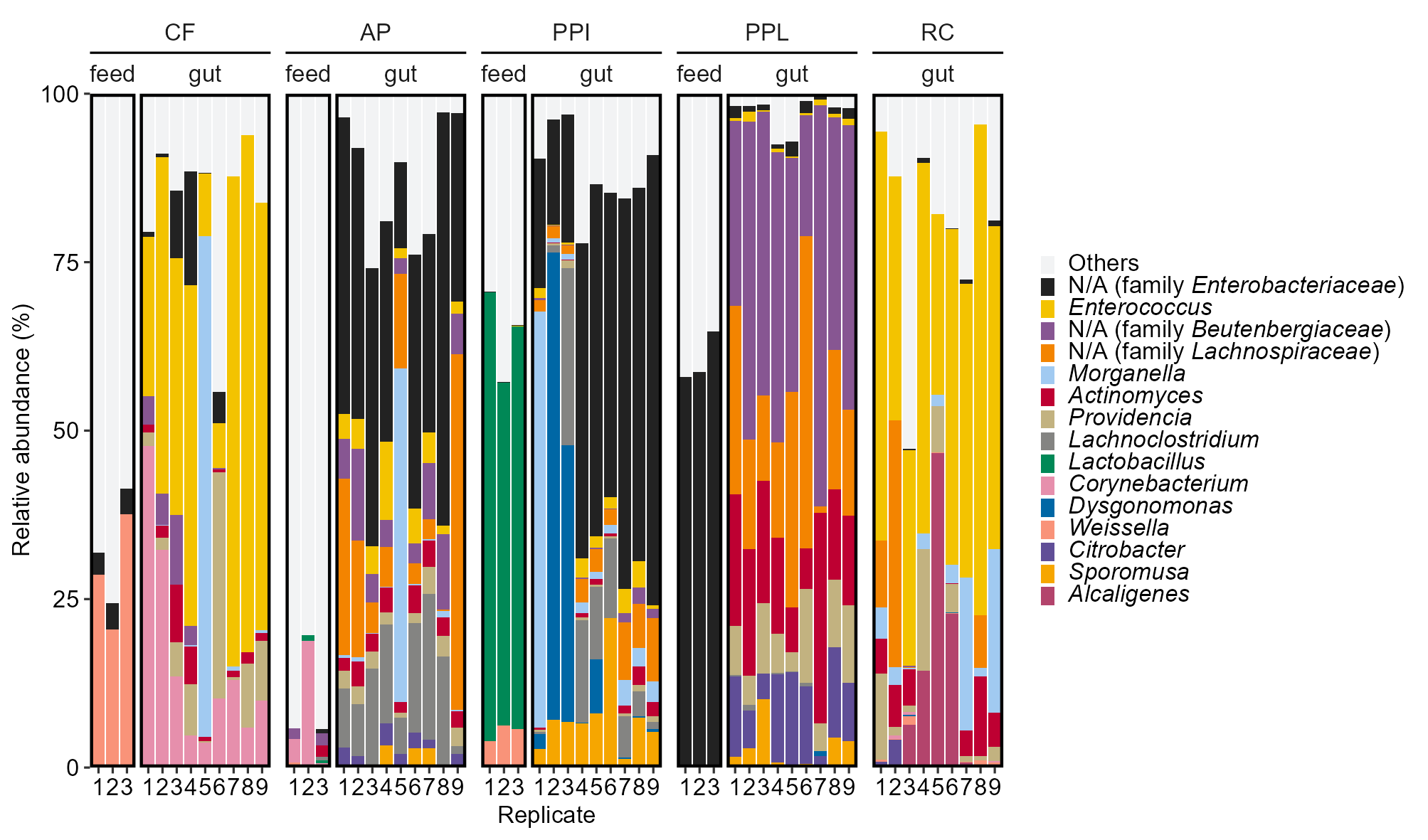
Figure S2. Stacked column plot of bacterial community composition in feed and BSFL gut from chicken feed (CF), apple pomace (AP), potato pulp industrial-scale (PPI), potato pulp lab-scale (PPL), and rapeseed cake (RC) group based on 16S rRNA gene amplicon sequencing. The RC-feed samples were excluded. The relative abundances of amplicon sequence variant (ASV) counts are displayed at the genus level for all replicates. Genera with low relative abundance are grouped and shown as “Others”. Only the top 15 genera are shown, measured by the relative abundance per genus level for all samples. The relative abundances based on unrarefied data.


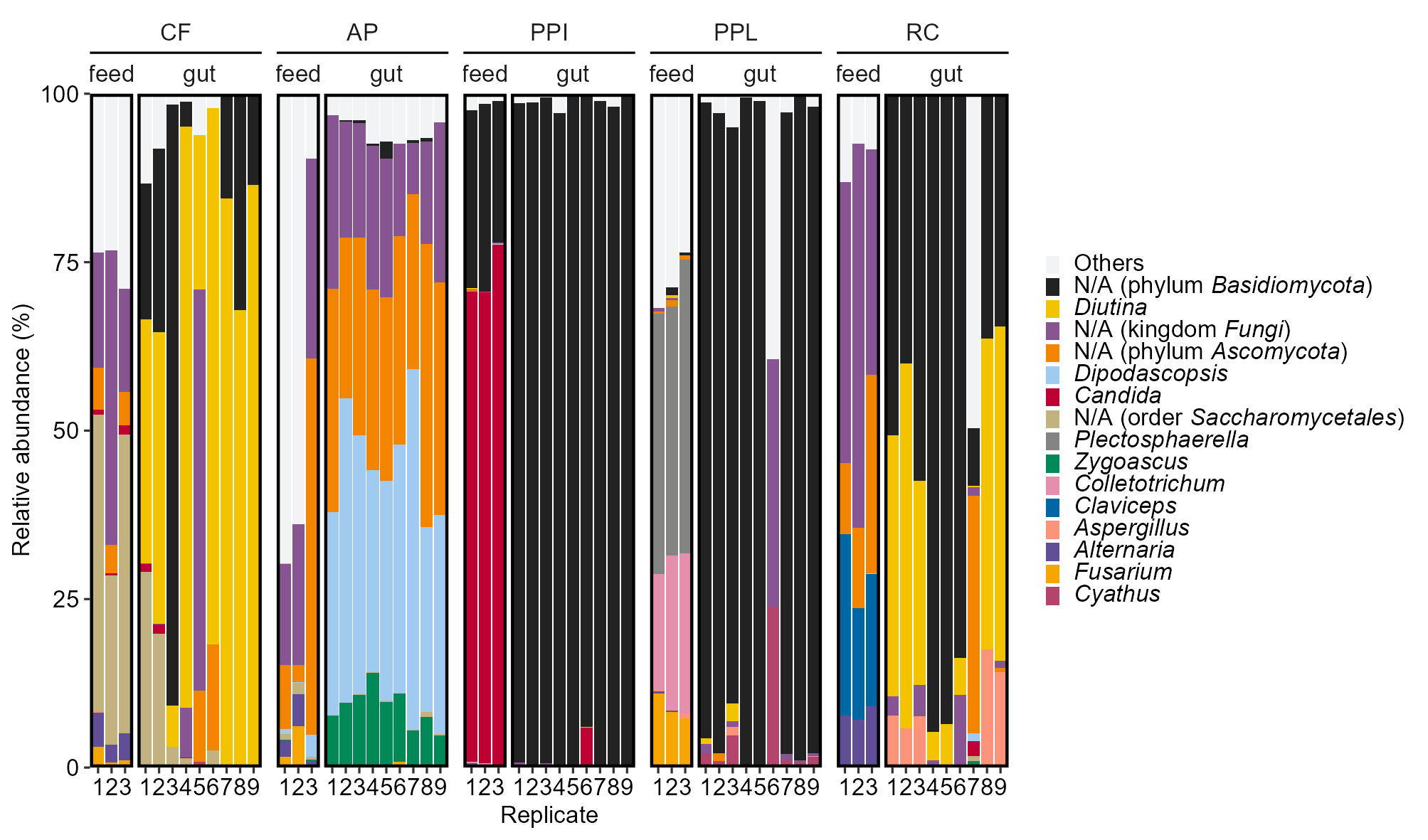


Figure S3. Stacked column plot of fungal community composition in feed and BSFL gut from chicken feed (CF), apple pomace (AP), potato pulp industrial-scale (PPI), potato pulp lab-scale (PPL), and rapeseed cake (RC) group based on ITS2 gene amplicon sequencing. The relative abundances of amplicon sequence variant (ASV) counts are displayed at the genus level for all replicates. Genera with low relative abundance are grouped and shown as “Others”. Only the top 15 genera of 218 are shown, measured by the relative abundance per genus level for all samples. The relative abundances are based on unrarefied data.
